# Benjie Foscablo: nursing in the pandemic

**DOI:** 10.2471/BLT.22.030422

**Published:** 2022-04-01

## Abstract

Benjie Foscablo talks to Gary Humphreys about the pressures of nursing in the pandemic, why nurses are leaving the profession, and the urgent need to improve nurses’ pay and conditions.

*Q: What drew you to the profession of nursing?*

A: Initially, it was something that I thought would help my family. When I was a kid, whenever we took a family member to a public hospital, I was shocked by the treatment we received. I felt that the nurses treated us very badly because we didn’t have money. Because of that experience, I said to myself, “You need to become a nurse. You need to know how to take care of your family members. Because how else are they going to get the care they need?” I decided that whatever it took, however much it cost – and getting a Bachelor of Science in nursing is expensive in the Philippines – I would do it. So, at first, I fought for my family, I fought for my parents and my siblings. Later on, as it became my career and my passion, I fought for my patients.

*Q: And yet you just walked away from nursing in hospitals. Can you talk about that decision?*

A: I took the decision to stop working in hospitals in December 2021 when it looked like the pandemic was slowing down. I said to myself, “I have done my part”. Of course, since then we have seen a fourth wave in the Philippines, so the pandemic is clearly not over. I am still working in the health sector, giving COVID-19 vaccinations in occupational health settings, but it is true that I am thinking about doing something else – working abroad, perhaps, or working in advocacy, where I can earn a decent living and conditions are not so stressful. I might even change profession. A lot of my peers have done the same thing, and it is a phenomenon that is happening worldwide. The pandemic has brought many health-system issues to the surface and the working conditions of health professionals is one of them. Nurses have received a lot of praise and applause, but the truth is that in most countries they continue to work in appalling conditions and for low wages. As the union representative at my hospital, I worked hard to change things, but much remains to be done.

*Q: Where did you work?*

A: At St Luke’s Medical Center, a private, 500-plus-bed, tertiary hospital in Taguig City, which is a part of Metropolitan Manila. I was there for nearly three years, starting out in the post-anaesthesia care and cardiovascular units. Then I was reassigned to a dedicated COVID-19 intensive care unit (ICU) which was set up to cope with the influx of cases in March of 2020.

“The pandemic has brought many health-system issues to the surface.”

*Q: What was it like to be put into the front line of the COVID-19 response?*

A: It was a little overwhelming, at first. All the patients we saw had the virus and a lot were elderly or had other illnesses and came in already in respiratory distress. So, there was a lot of anxiety about the patients, about fluctuating vital signs, people crashing, people dying. The level of activity was intense. On top of the usual tasks like preparing oral and intravenous medications, monitoring intake and outputs and titrating medications, we were doing all the prepping and monitoring related to oxygen treatment, getting patients ready for intubation, checking the mechanical ventilator settings, dealing with secretions in patients’ endotracheal tubes. We were also dealing with a lot of patients requiring resuscitation. We were quite stretched, with 8 to 10 nurses managing 15 COVID-19 beds. In a critical ICU you ideally have one nurse per bed, but unfortunately at times we needed to stretch the staffing ratios to one nurse to two or even three beds. Luckily, the doctors pitched in to help. Sometimes they were suctioning the endotracheal tubes to clear secretions while we were doing other tasks like getting medicines ready.

*Q: Were you concerned about getting infected yourselves?*

A: We were all frightened of the virus at first, and worried about getting infected and then spreading it to our families. A lot of us slept at the hospital to avoid that happening.

*Q: Did you have the personal protective equipment (PPE) you needed?*

A: We did, which was great, but of course PPE is itself challenging. When we were in the patient cubicles, we had to wear a full protective “bunny suit” and isolation gown, and then there were the goggles, a surgical mask on top of the N95 mask, a face shield and double gloves. We were glad to have it, but you can imagine what wearing all that was like for a 12-hour shift or when doing cardiopulmonary resuscitation, sometimes several times a day.

*Q: How many patients did you lose?*

A: I would say about half. Which is crazy to think about.

*Q: How hard was it having so many people die?*

A: Hard. It was especially hard for us to see patients dying without someone at their side, without any of their family by their side. We did our best to set up video calls, but it was not the same and often we were holding their hand when they died. And, you know, you’re being a nurse and you’re in this busy ICU but you’re also adding a role of being a family member, trying make them understand that their family loves them and that they will be missed. So, that was very hard. And resuscitation itself is hard. I mean, physically hard. I had one patient that we worked on for two hours. I still think about him. He was young, just 35 years old, and he had the strength to fight the virus, but he just wasn’t reacting to any of the management, and he couldn’t breathe and then his heart stopped. But we didn't give up because it seemed like there was still a chance. I injured my back that day.

*Q: How did you manage that kind of experience psychologically? Did it affect your sleep?*

A: You know, one of the advantages of total exhaustion is that you can sleep. Or at least I could. We were supposed to work eight-hour shifts, but we often worked 12 hours and sometimes even 16 hours. After a day like that, you sleep, whether it’s at the hospital or at home. You take a bus, you get home, you take a shower, and you sleep. Because you’re tired, but also because in six hours you need to wake up and go back in again.

*Q: Are those kinds of hours normal in the private sector?*

A: The pandemic made the situation worse, but long hours are common. The private sector labour standard is for a 40-hour week, but because of staff shortages most nurses in the private sector work an average of 60 hours per week. The public sector has similar rules, but they are enforced more, and you also have paid overtime and term offset rules, meaning that if you do go beyond your regular hours in a day, you can have additional time off. Pay is also worse in the private sector, with nurses earning around 537 Philippine pesos (₱) a day (roughly US$ 10), the minimum wage in Metropolitan Manila. In the public sector, an entry-level nurse earns around ₱ 33 000 (US$ 670) per month.

“In most countries [nurses] continue to work in appalling conditions.”

*Q: Were you never tempted to work in the public sector?*

A: Of course, but unless you have connections, you’re not going to be considered for a position, and even if you manage to get a place have to wait several years to get a proper contract with all the associated benefits. I also want to point out that there have also been issues with the allowances and benefits that had been agreed on and were supposed to help nurses during the pandemic, including a special risk allowance. These benefits have not been paid.

*Q: How many nurses are leaving the profession?*

A: According to the Private Hospitals Association of the Philippines, since the start of the pandemic, roughly 4 of 10 private hospital nurses have resigned. I’m not sure how credible that is but I know we lost around 200 people at St Luke’s and some of those people are thinking about going abroad.

*Q: The government of the Philippines has long had a policy of training health workers and supporting them in their pursuit of careers abroad. Has this created specific challenges at home?*

A: A lot of skilled people leave. This is a fact and of course the pandemic has increased opportunities with high-income countries calling for more nurses. The Philippine government actually banned nurse expatriations at the beginning of the pandemic and then introduced a cap of 5000 in 2021, but nurses continue to go abroad. The real problem is not losses to overseas countries, it is that people just leave the profession because the pay and conditions are so bad. There are big problems that need to be resolved in the domestic health-care system, not a lack of nurses but a lack of resources to support them, including the resources needed for decent pay and conditions. The pandemic has exacerbated the challenges. A lot of Filipinos have died in the pandemic without even going to the hospital. There is a huge problem of access. In some ways conditions haven’t changed so much since I was a kid. The needs of the poor are not being met. We are supposed to be moving towards universal health coverage, but we cannot achieve that if we don't invest in the health workers that deliver health services. So, I hope the government will invest in our people, our front-liners. They need to value their health professionals, and make it possible for them to live with the dignity and respect they deserve.

